# Lysophosphatidic acid and its receptors LPA_1_ and LPA_3_ mediate paclitaxel-induced neuropathic pain in mice

**DOI:** 10.1186/1744-8069-10-71

**Published:** 2014-11-19

**Authors:** Hitoshi Uchida, Jun Nagai, Hiroshi Ueda

**Affiliations:** Department of Pharmacology and Therapeutic Innovation, Nagasaki University Graduate School of Biomedical Sciences, 1-14 Bunkyo-machi, Nagasaki, 852-8521 Japan

**Keywords:** Paclitaxel, Lysophosphatidic acid, Neuropathic pain, MALDI-TOF-MS, Phospholipase A_2_, Spinal cord

## Abstract

**Background:**

Paclitaxel, which is widely used for the treatment of solid tumors, causes neuropathic pain via poorly understood mechanisms. Previously, we have demonstrated that lysophosphatidic acid (LPA) and its receptors (LPA_1_ and LPA_3_) are required for the initiation of peripheral nerve injury-induced neuropathic pain. The present study aimed to clarify whether LPA and its receptors could mediate paclitaxel-induced neuropathic pain.

**Results:**

Intraperitoneal administration of paclitaxel triggered a marked increase in production of LPA species (18:1-, 16:0-, and 18:0-LPA) in the spinal dorsal horn. Also, we found significant activations of spinal cytosolic phospholipase A_2_ and calcium-independent phospholipase A_2_ after the paclitaxel treatment. The paclitaxel-induced LPA production was completely abolished not only by intrathecal pretreatment with neurokinin 1 (NK1) or *N*-methyl-_D_-aspartate (NMDA) receptor antagonist, but also in LPA_1_ receptor-deficient (*Lpar1*^−/−^) and LPA_3_ receptor-deficient (*Lpar3*^−/−^) mice. In addition, the pharmacological blockade of NK1 or NMDA receptor prevented a reduction in the paw withdrawal threshold against mechanical stimulation after paclitaxel treatments. Importantly, the paclitaxel-induced mechanical allodynia was absent in *Lpar1*^−/−^ and *Lpar3*^−/−^ mice.

**Conclusions:**

These results suggest that LPA_1_ and LPA_3_ receptors-mediated amplification of spinal LPA production is required for the development of paclitaxel-induced neuropathic pain.

## Background

Peripheral neuropathic pain is produced by multiple etiologies, including physical trauma, metabolic diseases, viral infections, and chemotherapeutic agents [1, 2]. Paclitaxel (Taxol®) is one of the most widely used chemotherapeutic agents for various types of solid tumors such as breast, ovarian, and lung cancers. The treatment with paclitaxel is often accompanied by neuropathic pain, which is a major dose-limiting adverse effect and impairs the quality of life in patients [3, 4]. The abnormal pain can be developed as early as day 1 in patients [5] and several hours in animals [6] after initial treatment, and last for months to years after repeated administrations [4]. Until now, there is a lack of effective treatment for paclitaxel-induced neuropathic pain owing to the poor understanding of its molecular mechanisms.

Previously, we have clarified that lysophosphatidic acid receptor (LPA_1_) signaling initiates peripheral nerve injury-induced neuropathic pain and its underlying mechanisms, including demyelination [7]. Also, a single intrathecal injection of LPA evokes abnormal pain that mimics nerve injury-induced neuropathic pain, in terms of its pain phenotype, pharmacological characterization, and biochemical alterations [7–10]. Regarding the biosynthesis of LPA in the spinal cord, simultaneous intense stimulation with neurokinin 1 (NK1) and *N*-methyl-_D_-aspartate (NMDA) receptors activates phospholipase A_2_ to produce lysophosphatidylcholine (LPC), which is then converted to LPA by extracellular autotaxin enzyme [11]. Interestingly, intrathecally administrated LPA is capable of causing amplification of spinal LPA production [12, 13]. Furthermore, nerve injury causes amplification of spinal LPA production via LPA_1_ and LPA_3_ receptors [13], and, accordingly, nerve injury-induced neuropathic pain is absent in mice that are deficient in LPA_1_ or LPA_3_ receptors [7, 13]. These facts have suggested that LPA_1_ and LPA_3_ receptors-mediated amplification of spinal LPA production is required for the induction of neuropathic pain after nerve injury. However, whether LPA and its receptors could contribute to different types of peripheral neuropathic pain, including chemotherapy-induced neuropathic pain, remains to be determined. The present study showed that paclitaxel triggered LPA_1_ and LPA_3_ receptors-mediated amplification of spinal LPA production, by using matrix-assisted laser desorption/ionization time-of-flight mass spectrometry (MALDI-TOF-MS) with phosphate-capture molecule, Phos-tag. Furthermore, we found that paclitaxel-induced mechanical allodynia was robustly abolished in mice deficient in LPA_1_ or LPA_3_ receptors.

## Methods

### Animals

Male C57BL/6J mice (TEXAM corporation, Nagasaki, Japan) and homozygous mutant mice for the LPA_1_ [14] and LPA_3_ [15] receptor genes (*Lpar1*^*−/−*^ and *Lpar3*^*−/−*^) were used. These mutant mice were kindly provided by Prof. Jerold Chun (The Scripps Research Institute, La Jolla, USA). Mice used in this study weighed 20–24 g. They were kept in a room maintained at 21 ± 2°C and 55 ± 5% relative humidity with a 12 h light/dark cycle, and had free access to a standard laboratory diet and tap water. All procedures used in this work were approved by the Nagasaki University Animal Care Committee, and complied with the fundamental guidelines for the proper conduct of animal experiments and related activities in academic research institutions under the jurisdiction of the Ministry of Education, Culture, Sports, Science and Technology, Japan.

### Drug treatments

Paclitaxel (Taxol®) was kindly provided by Bristol-Myers Squibb Co. (NY, USA). This drug was dissolved in Cremophor EL and ethanol (1:1, v/v), and then diluted by physiological saline just before administration. A single or repeated intraperitoneal injection of paclitaxel (4 mg/kg) on 4 alternate days (day 0, 2, 4, and 6; cumulative dose of 16 mg/kg) was carried out. MK-801 was purchased from Sigma-Aldrich Co. (St. Louis, MO, USA), while CP-99994 was generously provided by Pfizer Inc. (NY, USA). These two inhibitors were dissolved in artificial cerebrospinal fluid (aCSF; 125 mM NaCl, 3.8 mM KCl, 1.2 mM KH_2_PO_4_, 26 mM NaHCO_3_, 10 mM glucose, pH7.4). CP-99994 (10 nmol/5 μl) and MK-801 (10 nmol/5 μl) were intrathecally administrated 30 min prior to paclitaxel injection. The intrathecal injection was given into the space between spinal L5 and L6 segments, according to the method of Hylden and Wilcox [16].

### Phospholipase A_2_ activity assays

According to the manufacturer’s protocol and our previous studies [13, 17], the activities of cytosolic phospholipase A_2_ (cPLA_2_) and calcium-independent phospholipase A_2_ (iPLA_2_) in the dorsal half of spinal cord were assessed by using cPLA_2_ assay kit (Cayman Chemicals, Ann Arbor, MI, USA). To assess cPLA_2_ activity, bromoenol lactone, a specific iPLA_2_ inhibitor [18], was added to the assay buffer. To measure the activity of iPLA_2_, sample was incubated with the substrate arachidonoyl thio-phosphorylcholine in modified Ca^2+^-free buffer (4 mM EGTA, 160 mM HEPES pH 7.4, 300 mM NaCl, 8 mM Triton X-100, 60% glycerol, 2 mg/ml of bovine serum albumin). The activity of PLA_2_ was defined as the percentage of the control activity as follows: paclitaxel-treated tissues (absorbance/mg of protein)/normal tissues (absorbance/mg of protein) × 100.

### Extraction of LPA and MALDI-TOF-MS analysis

LPA was extracted from the unilateral dorsal half of the lumber (L4-L6) spinal cord (6.15 mg tissue weight), as reported previously [13, 19, 20]. The final sample was dissolved in 50 μl of methanol containing 0.1% aqueous ammonia for MALDI-TOF-MS analysis. One μl sample was spotted on a MALDI plate (Bruker Daltonics, Inc., CA, USA). Immediately, 1 μl of 2’,4’,6’-trihydroxyacetophenone monohydrate solution (10 mg/ml in acetonitrile) was layered on the mixture as matrix solution. After drying, the sample was applied to Ultraflex-I^™^ TOF/TOF systems (Bruker Daltonics, Inc., CA, USA). Mass spectrometry was performed in the positive mode, using an accelerating voltage of 25 kV. The laser was used at energy of 30-50% (3.0-5.0 μJ) and a repetition rate of 10-Hz. The mass spectra were calibrated externally using Peptide calibration standard (Bruker Daltonics, Inc., CA, USA). Each spectrum was produced by accumulating data from 150 or 300 consecutive laser shots. Standard of 18:1-LPA was purchased from Sigma-Aldrich Co. (St. Louis, MO, USA), while standards of 16:0-, 17:0- and 18:0-LPA were obtained from Doosan Serdary Research Laboratories (London, ON, Canada).

### Nociception test

The mechanical paw pressure test was carried out, as described previously [7]. Briefly, mice were placed in a plexiglass chamber on a 6 × 6 mm wire mesh grid floor and allowed to acclimatize for a period of 1 h. A mechanical stimulus was then delivered onto the middle of the plantar surface of the animal using a Transducer Indicator (Model 1601; IITC Inc., Woodland Hills, CA, USA). The pressure needed to evoke a flexor response was defined as the pain threshold. A cut-off pressure of 20 g was set to avoid tissue damage.

### Statistical analysis

Statistical analysis was evaluated using the Dunnett’s test or a one-way ANOVA with Tukey-Kramer multiple comparison *post hoc* analysis. The criterion of significance was set at *p* <0.05. All results are expressed as mean ± SEM.

## Results

### Paclitaxel-induced LPA production and activations of iPLA_2_ an cPLA_2_ enzymes in the spinal dorsal horn

**Figure 1:**
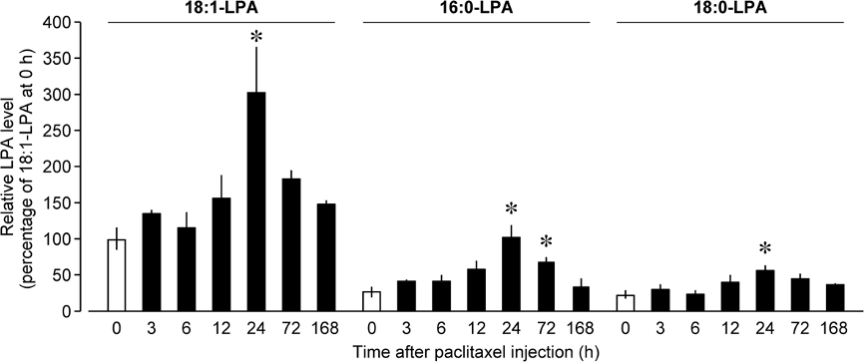
**Increase in spinal LPA level after paclitaxel injection.** Time courses of 18:1-LPA, 16:0-LPA, and 18:0-LPA levels in the spinal dorsal horn after the intraperitoneal injection of paclitaxel (4 mg/kg) were assessed by using MALDI-TOF-MS with Phos-tag. Data represent means ± SEM from experiments using 3–4 mice. *p < 0.05, versus corresponding 0 h.

Firstly, we assessed whether paclitaxel could induce LPA production in the spinal dorsal horn. In order to measure the levels of LPA species (18:1-, 16:0-, and 18:0-LPA), we performed quantitative MALDI-TOF-MS method with phosphate-capture molecule, Phos-tag, as reported previously [13, 19–21]. According to the ratios of ion-peak intensities with each standard of LPA species to that with internal standard 17:0 LPA (0.2 nmol), we have already validated the linearity of each calibration curve over the concentration range of 0.1-2.0 nmol [13]. Based on the calibration curves, the concentrations of LPA species were determined by measuring the ion-signals at *m/z* 997, 1023, and 1025, corresponding to 16:0-, 18:1-, and 18:0-LPA, respectively. As shown in Figure 1, the level of 18:1-LPA, which preferentially activates LPA_1_ and LPA_3_ receptors to initiate nerve injury-induced neuropathic pain [13], was gradually increased in the spinal dorsal horn after intraperitoneal injection of paclitaxel (4 mg/kg) and peaked at 24 h post-injection, followed by decline at 72 h post-injection. Similar alterations were also seen in the 16:0- and 18:0-LPA levels (Figure 1).

**Figure 2:**
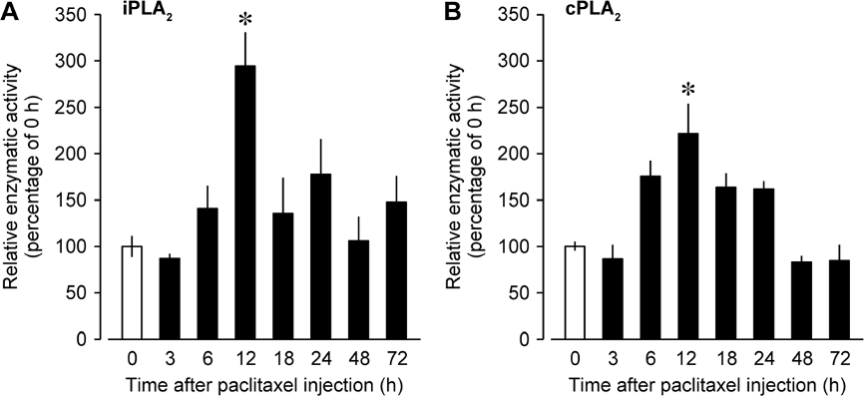
**Activations of iPLA**_**2**_ **and cPLA**_**2**_ **enzymes after paclitaxel injection. (A** **and** **B)** Activations of spinal iPLA_2_ **(A)** and cPLA_2_ **(B)** were measured by using iPLA_2_ and cPLA_2_ assays at indicated time points after the paclitaxel injection. Data represent means ± SEM from experiments using 3–5 mice. *p < 0.05, versus corresponding 0 h.

Our previous studies have demonstrated that iPLA_2_ and cPLA_2_ mediate the production of LPC, a precursor of LPA [11, 22, 23], in the spinal cord [11, 13, 17]. Here, we therefore carried out iPLA_2_ and cPLA_2_ assays to test whether paclitaxel could activate spinal iPLA_2_ and cPLA_2_ enzymes. The enzymatic activities of iPLA_2_ and cPLA_2_ in the spinal dorsal horn were significantly elevated at 12 h after the paclitaxel injection, followed by decline to the control levels at 48 h post-injection (Figure 2A and B).

### Involvement of substance P and glutamate receptors in paclitaxel-induced LPA production

**Figure 3:**
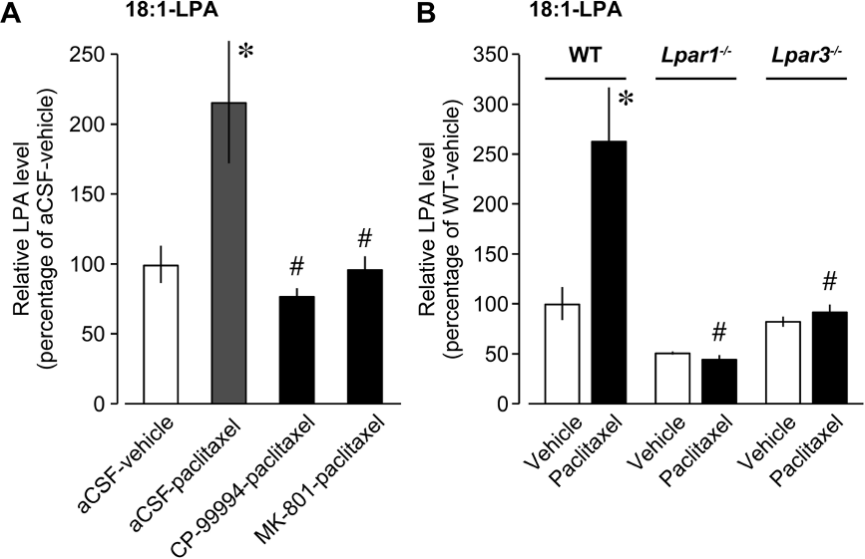
**Blockade of paclitaxel-induced LPA production.** Level of 18:1-LPA in the spinal dorsal horn at 24 h after the injection of paclitaxel or vehicle was measured by using MALDI-TOF-MS with Phos-tag. **(A)** CP-99994 (10 nmol), MK-801 (10 nmol), or aCSF was intrathecally injected at 30 min prior to the paclitaxel injection. Data represent means ± SEM from experiments using 3 mice. *p < 0.05, versus aCSF-vehicle-treated mice; ^#^ *p* < 0.05, versus aCSF-paclitaxel-treated mice. **(B)** Wild-type (WT), *Lpar1* ^−/−^, and *Lpar3* ^−/−^ mice were used to evaluate LPA level at 24 h after paclitaxel administration. Data represent means ± SEM from experiments using 3–5 mice. *p < 0.05, versus vehicle-treated WT mice; ^#^ *p* < 0.05, versus paclitaxel-treated WT mice.

It has been shown that excitatory neurotransmitters, substance P (SP) and glutamate, cooperatively evoke LPA production in the spinal cord [11]. In order to clarify the involvement of these factors in the paclitaxel-induced LPA production, mice were intrathecally pretreated with CP-99994 (10 nmol/5 μl), an NK1 antagonist, or MK-801 (10 nmol/5 μl), an NMDA receptor antagonist, at 30 min prior to the paclitaxel treatment. MALDI-TOF-MS analysis revealed that paclitaxel-induced production of 18:1-LPA was completely blocked by the pretreatment with CP-99994 or MK-801 (Figure 3A). Similarly, these inhibitors also abolished the paclitaxel-induced increases in 16:0- and 18:0-LPA levels (data not shown).

### Paclitaxel-induced amplification of LPA production via LPA_1_ and LPA_3_ receptors

Our previous studies have shown that LPA itself induces spinal LPA production [12, 13], and the amplification of LPA biosynthesis after peripheral nerve injury is abolished in *Lpar1*^*−/−*^ and *Lpar3*^*−/−*^ mice [13], indicating the critical involvement of LPA_1_ and LPA_3_ receptors. We therefore tested whether paclitaxel could trigger LPA_1_ and LPA_3_ receptors-mediated amplification of LPA production in the spinal cord. As shown in Figure 3B, paclitaxel-induced production of 18:1-LPA was absent in *Lpar1*^*−/−*^ and *Lpar3*^*−/−*^ mice. Also, *Lpar1*^*−/−*^ and *Lpar3*^*−/−*^ mice showed a lack of 16:0- and 18:0-LPA production after paclitaxel injection (data not shown).

### Blockade of paclitaxel-induced mechanical allodynia by NK1 and NMDA receptor antagonists

**Figure 4:**
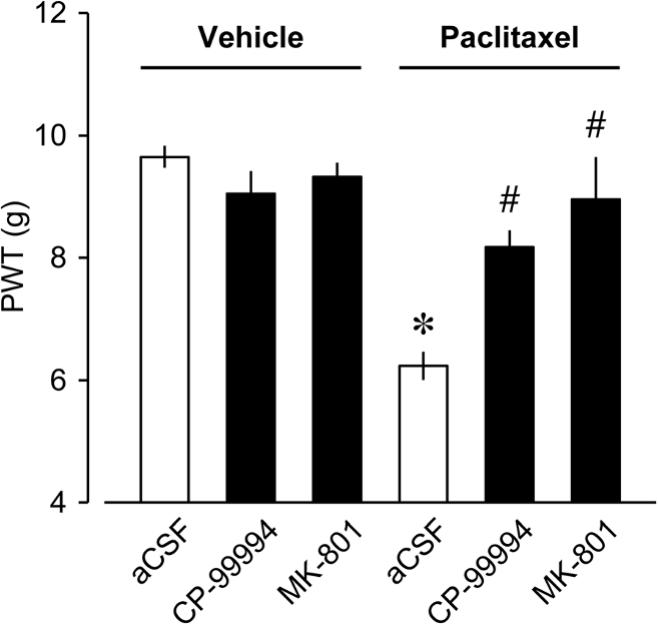
**Prevention of paclitaxel-induced mechanical allodynia by pharmacological blockade of NK1 or NMDA receptor.** Paclitaxel (4 mg/kg) was injected on 4 alternate days (day 0, 2, 4, and 6). CP-99994 (10 nmol), MK-801 (10 nmol) or aCSF was intrathecally injected at 30 min prior to the initial injection of paclitaxel. Mechanical paw withdrawal latencies (PWT, in g) were measured at 14 day after the initial paclitaxel injection, by using mechanical paw withdrawal test. Data represent means ± SEM from experiments using 6–10 mice. *p < 0.05, versus aCSF-vehicle-treated mice; ^#^ *p* < 0.05, versus aCSF-paclitaxel-treated mice.

Based on the findings that pharmacological blockade of NK1 and NMDA receptors inhibited paclitaxel-induced spinal LPA production (Figure 3A), we investigated whether SP and glutamate could mediate paclitaxel-induced neuropathic allodynia. The intraperitoneal treatments with paclitaxel (4 mg/kg) on 4 alternate days (day 0, 2, 4, and 6; cumulative dose of 16 mg/kg) significantly reduced the pain thresholds against mechanical stimuli at day 14 after the initial treatment (Figure 4). The paclitaxel-induced mechanical allodynia was completely blocked by the intrathecal pretreatment with CP-99994 (10 nmol/5 μl) or MK-801 (10 nmol/5 μl) (Figure 4). In contrast, CP-99994 and MK-801 had no effects on the mechanical pain thresholds in vehicle-treated mice (Figure 4).

### Involvement of LPA_1_ and LPA_3_ receptors in the development of paclitaxel-induced neuropathic pain

**Figure 5:**
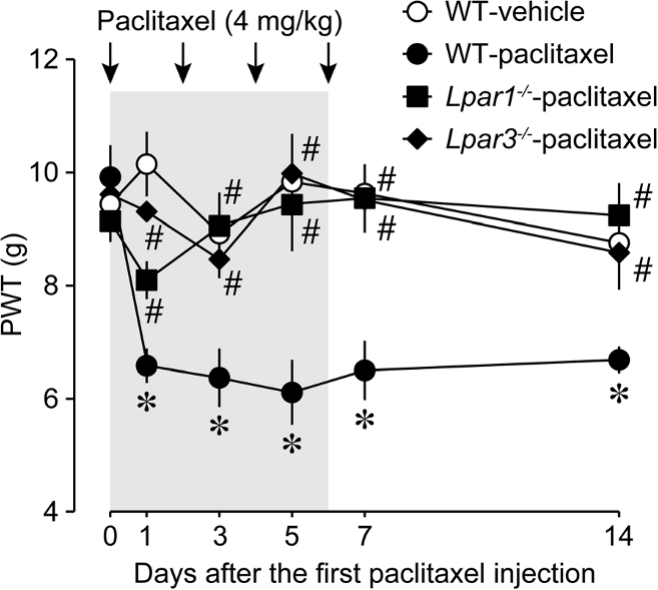
**Absence of paclitaxel-induced mechanical allodynia in LPA**_**1**_ **and LPA**_**3**_ **knockout mice.** Time course of mechanical paw withdrawal latencies (PWT, in g) after intraperitoneal injections of paclitaxel on 4 alternate days (day 0, 2, 4, and 6) in wild-type (WT), *Lpar1* ^*−/−*^, and *Lpar3* ^*−/−*^ mice. Mechanical pain thresholds were evaluated by using mechanical paw withdrawal test. Data represent means ± SEM from experiments using 4–6 mice. *p < 0.05, versus vehicle-treated WT mice; ^#^ *p* < 0.05, versus paclitaxel-treated WT mice.

To test whether LPA receptors could participate in paclitaxel-induced neuropathic pain, we used mice deficient in LPA_1_ or LPA_3_ receptors, both of which are required for the development of peripheral nerve injury-induced neuropathic pain [7, 13]. The paclitaxel treatments produced mechanical allodynia as early as day 1 after the first injection in wild-type animals (Figure 5). Intriguingly, the paclitaxel-induced mechanical allodynia was absent in *Lpar1*^*−/−*^ and *Lpar3*^*−/−*^ mice (Figure 5), suggesting the critical contribution of LPA_1_ and LPA_3_ receptors to the development of paclitaxel-induced neuropathic pain.

## Discussion

There is emerging evidence that LPA mediates not only peripheral nerve injury-induced neuropathic pain [7], but also other chronic pain, such as ischemia-induced central neuropathic pain [24] and bone cancer pain [25]. Here, we focused on the paclitaxel-induced neuropathic pain that can be observed in both clinic and animal studies [3]. The most important finding of the present study is that paclitaxel-induced spinal LPA production and the development of neuropathic pain were absent in *Lpar1*^*−/−*^ and *Lpar3*^*−/−*^ mice. This suggests, for the first time, the critical involvement of LPA and its receptors LPA_1_ and LPA_3_ in paclitaxel-induced neuropathic pain, a predictable adverse effect.

Our previous report has shown that peripheral nerve injury activates spinal iPLA_2_ and cPLA_2_ at 1 h post-injury, followed by spinal LPA production at 3 h post-injury [17]. Regarding mechanisms for the LPA biosynthesis, simultaneous stimuli of excitatory neurotransmitters, SP and glutamate, are capable of evoking LPA production in the spinal cord slices [11]. Indeed, pharmacological blockade of NK1 or NMDA receptor completely inhibits injury-induced iPLA_2_ and cPLA_2_ activation and LPA production [13]. In the present study, we found that paclitaxel caused a significant increase in spinal iPLA_2_ and cPLA_2_ activities at 12 h post-injection, followed by spinal LPA production at 24 h post-injection. In addition, the pretreatment with intrathecal NK1 or NMDA receptor antagonist completely blocked the paclitaxel-induced LPA production. These findings prompted us to hypothesize that paclitaxel could activate spinal iPLA_2_ and cPLA_2_, possibly via the increase in SP and glutamate levels, thereby leading to LPA production. In this context, paclitaxel, which poorly penetrates blood–brain-barrier, is known to accumulate in the dorsal root ganglion [26–28], indicating that paclitaxel could alter the functions of primary afferents to cause peripheral and central sensitization. For instance, there are reports showing that paclitaxel induces SP release from cultured primary afferent neurons [29, 30]. Moreover, paclitaxel has been found to cause a down-regulation of glial transporters, such as glutamate-aspartate transporter and glutamate transporter-1 in the spinal astrocyte as early as 4 h after paclitaxel injection [31], indicating that synaptic glutamate level would be increased in the spinal dorsal horn after paclitaxel administration. These mechanisms might explain the reasons why the paclitaxel-induced spinal iPLA_2_ and cPLA_2_ activation and subsequent LPA production are delayed as compared with the case of peripheral nerve injury.

Previously, we have demonstrated that a single intrathecal injection of LPA increases spinal LPA level [12, 13]. Also, nerve injury-induced production of LPA species is absent in *Lpar1*^*−/−*^ and *Lpar3*^*−/−*^ mice [13], indicating the involvement of LPA_1_ and LPA_3_ receptors in self-amplification of LPA production. Interestingly, 18:1-LPA, which preferentially activates LPA_1_ and LPA_3_ receptors, induces not only amplification of LPA production but also neuropathic thermal hyperalgesia [13]. In contrast, neither 16:0- nor 18:0-LPA triggers LPA production and neuropathic pain-like behavior [13]. Here, we showed that paclitaxel evoked a significant production of LPA species (18:1-, 16:0-, and 18:0-LPA), and such LPA production was absent in *Lpar1*^*−/−*^ and *Lpar3*^*−/−*^ mice. Therefore, it is logical to postulate that activations of LPA_1_ and LPA_3_ receptors, possibly by 18:1-LPA, are required for paclitaxel-induced amplification of LPA production. LPA can activate primary afferents, thereby evoking the release of SP from nerve endings via LPA_1_ receptor [32, 33]. Therefore, there is a possibility that LPA_1_ receptor might be involved in amplification of LPA production via the release of SP from presynaptic terminals in the spinal dorsal horn. Alternatively, LPA_1_ and LPA_3_ receptors are expressed in microglia [34, 35], indicating microglial LPA_1_ and LPA_3_ receptors might induce the release of biological factors that activate iPLA_2_ and cPLA_2_ to cause LPA production. One such candidate is interleukin-1β, which mediates LPA-induced LPA production via activations of iPLA_2_ and cPLA_2_ [36].

Tatsushima *et al.* reported that a single intrathecal injection of NK1 receptor antagonist alleviates the established mechanical allodynia after repeated treatments with paclitaxel [30], suggesting an involvement of SP in the maintenance of paclitaxel-induced neuropathic pain. Although NMDA receptor antagonist reverses the established mechanical allodynia after paclitaxel injections [37], inconsistent observation has been reported [38]. Therefore, the authors have mentioned that NMDA receptor activation is unlikely to have a major role in the maintenance of paclitaxel-induced neuropathic pain [37, 38]. On the other hand, the present study clearly showed that the intrathecal pretreatment with NK1 and NMDA receptor antagonists blocked paclitaxel-induced spinal LPA production and mechanical allodynia, suggesting that SP and glutamate are key mediators of the development of paclitaxel-induced neuropathic pain via causing LPA production. Furthermore, the paclitaxel-induced spinal LPA production and mechanical allodynia were absent in *Lpar1*^*−/−*^ and *Lpar3*^*−/−*^ mice. Collectively, these findings strongly suggest that spinally synthesized LPA, whose production is amplified via LPA_1_ and LPA_3_ receptors, participates in the pathogenesis of paclitaxel-induced neuropathic pain.

Regarding the molecular basis for induction of neuropathic pain, we have demonstrated that LPA evokes demyelination of sensory fibers in the dorsal root [7]. In addition, LPA also causes altered expression of pain-related molecules in the dorsal root ganglion and spinal dorsal horn [7]. Furthermore, gene profiling analysis has clarified that LPA initially up-regulates gene expression of ephrinB1, which contributes to LPA-induced neuropathic pain via activation of spinal NMDA receptor [39]. On the other hand, it has been proposed that multiple mechanisms, including mitochondrial dysfunction, altered gene expression, and glial activation, are implicated in the mechanisms for paclitaxel-induced neuropathic pain [27, 40]. Intriguingly, it seems that some of the mechanisms, such as demyelination and up-regulations of calcium channel α2δ-1 subunit, would be commonly observed in the neuropathic pain caused by paclitaxel and LPA [7, 41, 42]. Alternatively, a recent paper by Wright et al. has shown that phosphatidylinositol 4-phosphate 5 kinase type 1C has an important role in LPA-induced neuropathic pain [43], raising possibility that such lipid kinase might be involved in the LPA actions underlying paclitaxel-induced neuropathic pain. Further studies are required for the elucidation of mechanisms via LPA_1_ and LPA_3_ receptors underlying paclitaxel-induced neuropathic pain.

## Conclusions

The present study demonstrated that LPA_1_ and LPA_3_ receptors-mediated amplification of spinal LPA production is required for the development of paclitaxel-induced neuropathic pain. The molecular machineries underlying LPA production might serve as novel potential therapeutic targets in the prevention of paclitaxel-induced neuropathic pain.

## Abbreviations

aCSF: Artificial cerebrospinal fluid
cPLA_2_: Cytosolic phospholipase A_2_
iPLA_2_: Calcium-independent phospholipase A_2_
LPA: Lysophosphatidic acid
*Lpar1*^*−/−*^: LPA_1_ receptor-deficient mice
*Lpar3*^*−/−*^: LPA_3_ receptor-deficient mice
LPC: Lysophosphatidylcholine
MALDI-TOF-MS: Matrix-assisted laser desorption/ionization time-of-flight mass spectrometry
NK1: Neurokinin 1
NMDA: *N*-methyl-_D_-aspartate
SP: Substance P.
